# The 10-year incidence of hypertension across blood pressure categories in a population-based cohort in southwestern Sweden

**DOI:** 10.1186/s12872-021-02334-6

**Published:** 2021-10-29

**Authors:** Ulf Lindblad, Klara Lundholm, Jenny Eckner, Ying Li, Lennart Råstam, I. Margareta Hellgren, Bledar Daka

**Affiliations:** 1 School of Public Health and Community Medicine/Primary Care, Institute of Medicine, University of Gothenburg, PO Box 454, 405 30 Gothenburg, Sweden; 2 Department of Clinical Sciences Malmö, Lund University, Lund, Sweden

**Keywords:** Incident hypertension, High normal blood pressure, Prehypertension, Cohort study, Population survey

## Abstract

**Background:**

To explore the determinants of incident hypertension, and especially the impact of baseline blood pressure categories, in a representative Swedish population.

**Methods:**

A 10-year longitudinal study of residents aged 30–74. Blood pressures were measured and categorized according to ESH guidelines with optimal blood pressure < 120/80 mmHg, normal 120–129/80–84 mmHg, and high normal 130–139/85–89 mmHg. Incident hypertension was defined as ongoing treatment or three consecutive blood pressure readings ≥ 140/ ≥ 90 mmHg (one or both) at follow-up, while those with ≥ 140 and/or ≥ 90 mmHg at only one or two visits were labelled as unstable. After excluding subjects with hypertension, ongoing blood pressure lowering medication or a previous CVD event at baseline, 1099 remained for further analyses.

**Results:**

Sixteen (2.6%) subjects with optimal baseline blood pressure had hypertension at follow up. Corresponding numbers for subjects with normal, high normal and unstable blood pressure were 55 (19.4%), 50 (39.1%) and 46 (74.2%), respectively. Compared with subjects in optimal group those in normal, high normal and unstable blood pressure categories had significantly higher risk to develop manifest hypertension with odds ratios OR and (95% CI) of 7.04 (3.89–12.7), 17.1 (8.88–33.0) and 84.2 (37.4–190), respectively, with adjustment for age, BMI and family history for hypertension. The progression to hypertension was also independently predicted by BMI (*p* < 0.001), however, not by age.

**Conclusions:**

Subjects with high normal or unstable blood pressure should be identified in clinical practice, evaluated for global hypertension risk and offered personalized advice on lifestyle modification for early prevention of manifest hypertension and cardiovascular disease.

## Introduction

Hypertension is a common condition, strongly associated with cardiovascular mortality and morbidity [1, 2] and 2010 established as the leading cause behind the world’s global burden of disease [2, 3], and as recently reviewed and put into a context of causal determinants by Fuchs and Whelton [3]. The mechanisms behind essential hypertension are complex and yet not fully understood although both multiple biological and behavioural factors are known to be involved in hypertension development [4]. The prevalence of hypertension often increases with age [5], and earlier longitudinal studies also indicate that blood pressure levels late in life can be traced back to levels earlier in life, even in childhood [6]. Still, the development of hypertension with increasing age should not be considered inexorable if proper preventive actions are taken [3]. Correspondingly, subjects with high blood pressure, also within the normal range, run an increased risk of developing hypertension compared to subjects with lower blood pressure [3] and there also seems to be a sliding scale of elevated cardiovascular disease risk associated with blood pressures all the way down to a level of 115/75 mmHg [7]. European society of hypertension [8] defines *high normal blood pressure* as 130–139 mmHg systolic and/or 85–89 mmHg diastolic. Estimates of hypertension conversion rate vary, but earlier studies [9–11] suggest that 2–4 individuals out of 5 are at risk to convert to hypertension in 4–10 years depending on age and level of economic development in the country for the study. Therefore, while a presumptive cardiovascular risk factor in its own right, high normal blood pressure also constitutes a risk factor for future hypertension and has sometimes even been referred to as *prehypertension*. Thus, subjects of high normal blood pressure, especially in younger population strata, constitute a highly interesting and still rather poorly studied group.

Our study aim was accordingly to study the determinants for incident hypertension among a representative population sample of individuals mainly in their early middle-ages with focus on baseline blood pressure categories.

## Methods

### Study design and subjects

**Fig. 1:**
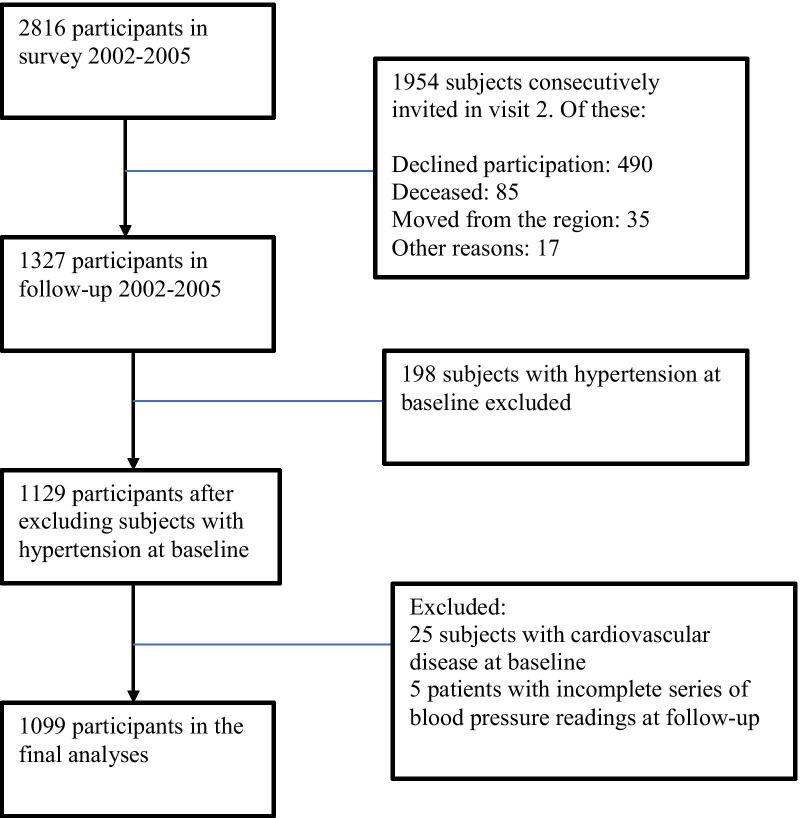
Overview of the Vara–Skövde cohort: study design and follow-up

The first visit of this prospective study, including 2816 individuals, was performed 2002–2005 in the two municipalities Vara and Skövde in the south-west of Sweden. All participants were randomly selected from the population census register, 30–74 years old, stratified by gender and five-year age groups with intentional oversampling (threefold) in the age group 30 to < 50 years as compared with those aged 50 years or older. Signed informed consent was obtained from all participants. A follow-up visit including 1327 representative individuals was performed between 2012 and 2014, 9.7 years after base-line with a participation rate of 66%. The same protocol as at baseline visit was used at the follow-up visit. Individuals diagnosed with hypertension or on anti-hypertensive medication at base-line were excluded in this study (n = 198), as were those with a previous CVD event (n = 25), and 5 participants lacking a complete series of blood pressure readings at follow-up, leaving 1099 individuals for the final analyses (Fig. 1).

### Anthropometric and blood pressure measurements

The participants were carefully examined by two specially trained nurses who measured body weight and height, waist-circumference, and blood pressure. Body weight was measured with participants wearing light clothing and no shoes, on a calibrated scale to the nearest 0.1 kg. Body height was measured without shoes to the nearest cm. Blood pressure was measured according to expert guidelines [8] in the right arm after a five minutes rest, the participant in a supine position with the cuff at heart level supported by a pillow and repeated twice with a one minute interval using the mean for statistical analyses. Tricuff™ was used for automatic adjustment of cuff size to arm circumference and the reading was done at the closest 2 mmHg. If blood-pressure was ≥ 140 mm Hg systolic and/or ≥ 90 mm Hg diastolic the participant was re-examined after one to two weeks and if still exceeding recommendations the examination was repeated a third time and if still ≥ 140/90 mm Hg the participant was diagnosed with hypertension [8] and referred to the health-care unit. Blood pressure was further categorized according to European expert guidelines [8] into optimal blood pressure, normal blood pressure, and high normal blood pressure respectively (Table 1). An additional category, *unstable* blood pressure, was further added, including subjects with a blood pressure measurement ≥ 140/90 mm Hg on one or two visits but not on three. If systolic and diastolic blood pressures were categorized differently the higher was applied.

**Table 1:** Blood pressure categories according to ESH/ESC guidelines 2018

Category	Systolic (mmHg)		Diastolic (mmHg)
Optimal	< 120	and	< 80
Normal	120–129	and/or	80–84
High normal	130–139	and/or	85–89
Grade 1 hypertension	140–159	and/or	90–99
Grade 2 hypertension	160–179	and/or	100–109
Grade 3 hypertension	≥ 180	and/or	≥ 110

When a subject’s systolic and diastolic blood pressures fall into different categories, the higher category is applied

Venous blood samples were drawn in the morning after an overnight fast and all participants performed an oral glucose tolerance test (OGTT). New diagnosis of diabetes mellitus was confirmed after two fasting plasma glucose values of ≥ 7.0 mmol/L, or one 2-h plasma glucose value of ≥ 11.1 mmol/L in an oral glucose tolerance test [12]. Insulin resistance was estimated based on the Homeostasis Model Assessment of insulin resistance (HOMA-ir): fasting insulin × fasting blood glucose/22.5 [13]. Plasma concentrations of lipids were analysed using standard methods.

The participants were interviewed by trained nurses concerning medical history and medication. All participants also completed questionnaires concerning psychosocial health, lifestyle, i.e., physical activity and smoking, stress and quality of life. The participants were asked to grade their leisure time physical activity the last year in a validated, four-graded questionnaire: “How physically active are you during your leisure time? (1) Sedentary leisure time: reading, watching television, stamp collecting or other sedentary activity; (2) Light leisure time physical activity: walking, cycling, or similar physical activity at least four hours per week; (3) Moderate leisure time physical activity: running, swimming, tennis, aerobic, heavier gardening, or similar activity during at least 2 h per week; (4) Heavy training or competitive sport in running, skiing, swimming, football, etc., performed regularly and several times a week” [14].

Information on level of education was also collected by the questionnaire and was further categorised as those with ≤ 9 years, 9–12 years, and > 12 years education. A family tree tracking the presence of hypertension among first degree relatives was created. For this study a family history of hypertension was defined as the occurrence among one or both parents and/or among the siblings. Alcohol consumption during the last month was estimated using a technic Time-Line Follow-Back systematically reviewing intake of beer, wine and liquor by frequency and quantities. An algorithm was then used to estimate gram alcohol consumed by time allowing also for the effect of binge drinking [15]. For these analyses we compared those with regular consumption of alcohol (g/week > 0) to non-consumers during the last 30 days. Further information on characteristics and analyses has been described in detail before [16].

### Statistical analyses

All statistical analyses were performed using the SPSS software package for Windows, version 24.0. Logistic regression was used to investigate association between baseline blood pressure categorical variable and the development of hypertension, with adjustment with continuous age, BMI at baseline and binary family history of hypertension. In addition, baseline blood pressure category was also used as a continuous variable in the regression to test the trend of association. In an extended model we added information on duration of education, type 2 diabetes, level of leisure time physical activity (LTPA), concentration of LDL cholesterol, and alcohol use as covariates.

Stratification of analyses for sex revealed no significant sex-differences, and when adjusting for age no significant interactions between sex and each of the other factors included in the multivariable analysis were significant (Data not shown).

## Results

Characteristics of the baseline population are presented in Table 2. Among the 1099 participants 625 (56.9%) had optimal blood pressure, 284 (25.8%) had normal blood pressure, 128 (11.6%) had high normal blood pressure and 62 (5.6%) had unstable blood pressure at the baseline survey. Mean follow-up time in this cohort was 9.7 years and during this period we observed 167 (15.2%) incident cases of hypertension during follow-up. Only 16 (2.6%) of 625 subjects with optimal baseline blood pressure converted to hypertension during follow up, while corresponding figures were 55 (19.4%) within normal, 50 (39.1%) with high normal, and 46 subjects (74.2%) with an unstable blood pressure, respectively.

**Table 2:** Characteristics of the study population at baseline

Characteristics	All n = 1099	
	Mean	SD
Age (years)	46.7	10.6
BMI (kg/m^2^)	26.2	3.8
LDL-cholesterol (mmol/L)	3.2	0.9
HDL-cholesterol (mmol/L)	1.3	0.3

	Median	Inter-quartile range
Triglycerides (mmol/L)	1.05	0.77–1.44
HOMAir (mmol*mU/L^2^)	1.13	0.76–1.71
Alcohol (g/week)	25.2	8.4–62.1

	Number	Per cent
Sex (male)	537	48.9
*Blood pressure category*		
Optimal	609	65.3
Normal	229	24.6
High normal	78	8.4
Unstable	16	1.7
Diabetes Mellitus type 2	21	1.9
Daily smoking	154	14
*Leisure time physical activity*		
Inactive	68	6.2
Little active	620	58.3
Moderately active	338	31.8
Heavily active	38	3.6
Family history of hypertension	441	40.8
*Education*		
≤ 9 years	271	25
9–12 years	428	40
> 12 years	380	35

*BMI* body mass index, *LDL* low-density lipoprotein, *HDL* high-density lipoprotein, *HOMA-ir* homeostatic model assessment of insulin resistance, *SD* standard deviation

Accordingly, normal, high normal and unstable baseline blood pressure were all significantly associated with the development of manifest hypertension compared to optimal blood pressure OR (CI), of 7.04 (3.89–12.7), 17.1 (8.88–33.0) and 84.2 (37.4–190), respectively (Table 3), test for trend *p* < 0.001. These results remained practically the same when also duration of education, leisure time of physical activity, type 2 diabetes, LDL-cholesterol and alcohol use were included in the regression (Table 3). The progression to hypertension was also significantly associated with BMI as shown in Table 3. However, age and a family history of hypertension did not contribute statistically significant to the incidence of hypertension in this model (Table 3). When Homa-ir was substituted for BMI in this model it also came out statistically significant (*p* < 0.001), however, the OR’s for the other covariates remained practically the same. Level of physical activity, reported alcohol intake, level of education and concentrations of LDL cholesterol was, however, not predictive of progression to manifest hypertension in corresponding multivariate models (data not shown).

**Table 3:** Determinants of incident hypertension over 10 years in the Vara/Skövde Cohort

Variables	OR	95% CI	p
*Covariates in model age, BMI, Family history of hypertension, BP category at baseline*			
Age	1.01	0.99–1.03	0.153
BMI	1.12	1.06–1.18	< 0.001
Family Hx hypertension	1.47	0.99–2.20	0.059
Blood pressure category at baseline			
Optimal	1		
Normal	7.04	3.89–12.7	< 0.001
High normal	17.1	8.88–33.0	< 0.001
Unstable	84.2	37.4–190	< 0.001
*Covariates in model age, BMI, Family history of hypertension, BP category at baseline, education, Type 2 diabetes, LDL Cholesterol, alcohol use*			
Age	1.01	0.99–1.04	0.393
BMI	1.11	1.05–1.17	< 0.001
Family history of hypertension	1.43	0.94–2.18	0.095
Blood pressure category at baseline			
Optimal	1		
Normal	6.58	3.62–12.0	< 0.001
High normal	16.0	8.21–31.2	< 0.001
Unstable	77.5	32.9–183	< 0.001
Education			
≤ 9 years	1		
9–12 years	0.95	0.55–1.64	0.853
> 12 years	0.69	0.38–1.25	0.222
Type 2 diabetes at baseline yes/no	2.51	0.86–7.33	0.092
Leisure time physical activity low/high	1.01	0.64–1.60	0.965
LDL cholesterol mmol L-1	1.05	0.81–1.35	0.722
Alcohol users vs non-users	1.13	0.64–1.99	0.677

Missing data: BMI (n = 1), family history of hypertension (n = 19), education (n = 20), leisure time physical activity (n = 35), LDL cholesterol (n = 2), alcohol consumption (n = 35)

*BMI* body mass index

## Discussion

Our results confirm the fact that high normal blood pressure by far constitutes the strongest risk factor for development of manifest hypertension, independent of age, BMI and family history of hypertension [5, 17, 18]. Furthermore, the risks associated with normal, high normal and unstable baseline blood pressure increase in a dose dependent manner compared to optimal blood pressure. While overweight constituted the second strongest finding regarding the transition from high normal blood pressure to manifest hypertension nor age or a family history of hypertension contributed significantly to the risk of incident hypertension.

In other cohort-studies with similar socio-economic development as ours, OR’s for conversion from high normal blood pressure to manifest hypertension were similar to our findings using optimal blood pressure as reference [3, 9, 11], while OR’s were correspondingly higher in unacculturated countries [10]. Unstable blood pressure at the baseline survey was associated with the highest risk of development of hypertension as the vast majority in this category were hypertensive at follow-up. Possibilities to compare this result with previous studies are limited due to the fact that the definition of unstable blood pressure is not generally acknowledged. However, our results emphasize the importance to follow-up subjects with unstable blood pressure due to their high risk to convert into hypertension.

In epidemiological surveys a diagnosis of hypertension is generally based on a single blood pressure measurement at the study visit. However, the blood pressure is known to vary in between readings and especially between different visits to a clinic, and repeated measurements are likely to reduce the misclassification associated with this phenomenon. In this study three consecutive measurements were required for the diagnosis of hypertension, thus simulating the procedures in a more clinical setting and accordingly defining a more valid target group. This procedure is likely to reduce the proportion identified with hypertension, however, in a previous publication from this cohort we showed that the prevalence of hypertension was only six per cent higher using only the first blood pressure measurement compared to strictly applying three consecutively high blood pressures [19]. Still, the reduced misclassification should increase the generalizability and the implications of our findings.

The blood pressure category in focus here, high normal blood pressure, has in some contexts been labelled prehypertension based on its increased risk of hypertension within near future [5, 17]. Actually, the latest expert guidelines in the US have included this segment of the blood pressure distribution in the diagnosis of hypertension [20]. While lifestyle modifications are the recommended intervention, also pharmacological treatment can be advocated when the global risk is estimated to be high. The association between overweight and the development of hypertension is well established before [21]. Hypertension is also known to cluster within families, even so family history has seldom been considered in previous studies [9, 22–24]. We found that a history of hypertension among first degree relatives was not associated with development of hypertension, however, it was close to significance (OR 1.47 (CI 0.99–2.20, *p* = 0.057)), and a type 2 error may be at hand. Therefore, we suggest that the clinical implications of heritability should be further studied in the future. Indeed, part of the effects of heritability might be expressed through the impact of baseline blood pressure categories as observed in this study.

The results obtained are in accordance with previous studies and confirm the fact that also a mild blood pressure elevation, already in middle age, constitutes a serious risk factor for future hypertension. CVD-risk grading tools, such as SCORE [25], are important instruments for cardiovascular disease- and death-risk calculation and can serve as support in approaching question of treatment initiation. We believe, however, that in order to make prevention of cardiovascular disease more effective, employing early prevention of hypertension is essential as that may in a large part reduce the vascular consequences usually attributed to aging and further limit the population burden of blood pressure-related CVD as expressed by Fuch and Whelton [3]. That age in itself is not the dominant factor is also illustrated by the increased risk of conversion from high normal blood pressure to manifest hypertension also generally seen among younger population strata [3, 9–11]. In fact, most participants in our cohort were aged 30–50 years at baseline and thus illustrates the development of hypertension from adulthood into early middle-ages before age-related chronic conditions take overhand. Results obtained from this study can be instantly implemented into clinical practice in primary care through doctors emphasizing current blood pressure and BMI as major factors when identifying subjects in need of preventive measures to reduce the risk of developing hypertension. The major importance of BMI is in accordance with general recommendations of the maintenance of a healthy body weight by means of attention to caloric intake and physical activity [26] and findings in previous interventions to reduce blood pressure using a strict diet on low sodium and the DASH-diet, respectively [27, 28]. The implications for clinical practice and strategies of prevention are obvious.

Among the strengths of this study are the representative population-based sampling and the comparably high participation rate, that confers a strong generalizability. It should be emphasized that our study population derives from two smaller communities, Vara with about 16,000 residents and Skövde with about 50,000 residents, and thus exposed to a higher prevalence of overweight and obesity as there is a strong gradient along the population density for these conditions as recently published by Hemmingsson et al. [29]. Other differences in lifestyle and in the environment should also be considered when comparing the outcome to that in bigger cities, however, the strong basic determinants should still be the same. Further strengths are the utilization of standard methods and the strict repeating of the same study protocol. Limitations of this study include the natural risk for inaccuracy regarding self-reported information and losses to follow-up. Furthermore, the study did not take into account diet and especially not salt consumption, an important factor in hypertension aetiology and blood pressure regulation, thus a potential residual confounder [27]. Though length of education was not a significant factor in the incidence of hypertension in our study other information on socioeconomic aspects could also have been valuable in the analysis.

In conclusion, high normal blood pressure is common in the general population and these individuals run a considerable risk of progression to manifest hypertension already in the middle-ages, as do individuals with one or two blood pressures within the hypertension range. They should be easy to identify among the flow of patients in primary care applying the present findings and proper intervention should be in accordance with general preventive strategies with a focus on diet and starting in young adults. However, controlled clinical trials comprising also cost benefit analyses are called for in this area.

## Data Availability

The datasets and/or analyses during the current study are available from the corresponding author on reasonable request.

## Abbreviations

BMI: Body mass index
CI: 95% Confidence interval
ESC: European Society of cardiology
ESH: European Society of hypertension
HDL: High-density lipoprotein
HOMA-ir: Homeostatic model assessment of insulin resistance
LDL: Low density lipoprotein
OR: Odds ratio
SD: Standard deviation
